# Genome sequence of *Pseudomonas silesiensis* strain G3, a member of a bacterial consortium capable of polyethylene degradation

**DOI:** 10.1128/mra.00138-24

**Published:** 2024-09-27

**Authors:** Beate Schneider, Friedhelm Pfeiffer, Daniela Remus, Mike Dyall-Smith, Hans-Jörg Kunte

**Affiliations:** 1 Division Biodeterioration and Reference Organisms, Bundesanstalt für Materialforschung und -prüfung, Berlin, Germany; 2 Computational Systems Biochemistry, Max-Planck-Institute of Biochemistry, Martinsried, Germany; 3 Biology II, Ulm University, Ulm, Germany; 4 Veterinary Biosciences, Melbourne Veterinary School, Faculty of Science, University of Melbourne, Parkville, Victoria, Australia; Wellesley College Department of Biological Sciences, Wellesley, Massachusetts, USA

**Keywords:** plastic degradation, polyethylene, consortium

## Abstract

A consortium of landfill bacteria including strain G3 can break down polyethylene, a long-lasting plastic that accumulates in the environment. The complete genome sequence of strain G3 was determined by PacBio and Nanopore sequencing and consists of three circular replicons. Genome-based classification assigned strain G3 to the species *Pseudomonas silesiensis*.

## ANNOUNCEMENT

*Pseudomonas silesiensis* G3 was isolated from a landfill (Niemegk-Neuendorf, 52°04′59″N, 12°41′59″E) and together with eight other isolates degrades polyethylene. A bacterial suspension from the landfill was diluted in soft agar and incubated in an iChip (1–4). The emerging microcolony of strain G3 was streaked on agar. Cells from one colony were grown in tryptic soy broth (28°C), washed, centrifuged, and stored (−80°C). Isolates were distinguished by microscopy, colony morphology, and 16S rRNA sequence. The primers (GAGTTTGATCMTGGCTCAG and GGYTACCTTGTTACGACTT) used for 16S rRNA analysis amplified a 1,405-bp fragment, which showed 100% sequence identity to, e.g., *Pseudomonas silesiensis* strain A3, accession CP014870.

Combining PacBio and Oxford Nanopore sequencing, the whole-genome sequence was determined. For Nanopore sequencing, DNA was isolated using a Marmur procedure (5), which comprises cell lysis with EDTA-lysozyme-RNaseA and SDS, DNA purification using Na chloride/chloroform:isoamyl alcohol, and DNA precipitation, drying, and resolving in Tris-EDTA. The DNA library was prepared without prior size selection (Kit SQK-RAD004, protocol_RSE_9046_v1_14Aug2019, Oxford Nanopore). Sequencing was performed on a MinION sequencer with a flongle flow cell (FLO-FLG001) using MinKNOW (v22.10.7). The sequencing resulted in 35,180 reads (N50 = 12.24 kb, discarding reads <200 nt), which were base-called using Guppy (v6.1.2).

For PacBio sequencing at Genewiz/Azenta (Leipzig, Germany), DNA was extracted (Qiagen Genomic-tip 100/G) from pelleted cells and sheared to ~10 kb (g-TUBE device) without size selection. DNA quality (Nanodrop, pulsed-field gel analysis) and quantity (Qubit 2.0) were assessed. A library (SMRTbell library-kit v2.0, PacBio) was prepared according to the manufacturer’s instructions and sequenced on a PacBio Sequel machine (6) using one SMRT cell (2–4). This generated 750,181 subreads (average length 2,687 nt; N50 = 3,035 nt; total bases = 2.0 Gb) with a 247-fold coverage. Coverage was reduced to 200-fold by random down-sampling, followed by processing with Canu (7) (v1.7, default settings), to output 52,533 reads (N50 = 4,416 nt).

Nanopore reads were assembled using Flye (v2.9.1) followed by polishing using Medaka (v1.5), which produced three circular contigs (7.0 Mb, 1.1 Mb, and 61 kb) with 50-, 70-, and 155-fold coverage. PacBio-fastq reads were mapped to the Nanopore contigs using Minimap2 (v2.44) within Geneious Prime (v2023.2.1, default settings), resulting in 27.4-, 28.4-, and 48.7-fold coverage (from largest to smallest). The PacBio reads were used to correct the Nanopore contigs and analyze DNA methylation (Table 1) using the idpSummary tool (v3.0) within SMRTLINK (v11.0) (8) and MultiMotifMaker (v1) (9).

**TABLE 1:** DNA methylation analysis^*a*^^,^^*b*^

Motif	Modification	Fraction	nDetected	nGenome	meanScore	meanIpdRatio	meanCoverage	objectiveScore
GTAANNNVNTTC	m6A	0.25	98	400	86.2	3.19	117.1	2,381.8
GAANBNNNTTAC	m6A	0.20	79	400	86.7	3.07	114.7	1,591.1

^*a*^ Methylation of adenosine (m6A) in the motif and its complement is underlined.

^*b*^ Approximately 20% of sites were modified.

Using only the Type-Strain Genome Server (TYGS) for taxonomic classification (10), strain G3 was assigned to the species *Pseudomonas silesiensis*, with a digital DNA–DNA hybridization value (*d*_4_) of 71.3% (95% confidence interval, 68.3%–74.2%) (11) to the type-strain *Pseudomonas silesiensis* A3 (GenBank accession: CP014870) (12). The genome of *Pseudomonas silesiensis* G3 consists of one chromosome (7,006,674 bp; 59.5% GC content), which was rotated manually using Emacs, a large plasmid (1,168,067 bp; 53.3% GC content) and a small plasmid (61,534 bp; 55.4% GC content). The large plasmid causes the difference in GC content (0.96%) compared to the type-strain. TYGS analysis of the chromosome alone minimized the difference to 0.04%. The genome was annotated at GenBank using PGAP v6.1 (13), identifying 7,589 protein-coding genes.

## Data Availability

The genome was deposited in GenBank under the BioProject accession number PRJNA1068290 and the BioSample accession number SAMN39584110. The nucleotide sequence accession numbers are CP143487 (chromosome), CP143488 (large plasmid), and CP143489 (small plasmid). The Sequence Read Archive (SRA) accession numbers are SRR27781161 and SRR27781162.
